# Rice Husk as Raw Material in Synthesis of NaA (LTA) Zeolite

**DOI:** 10.3390/molecules29184396

**Published:** 2024-09-16

**Authors:** Daniela Novembre, Domingo Gimeno, Lucia Marinangeli, Anna Chiara Tangari, Gianluigi Rosatelli, Michele Ciulla, Pietro di Profio

**Affiliations:** 1 Dipartimento di Ingegneria e Geologia, Università degli Studi “G. D’Annunzio”, Via dei Vestini 30, 66013 Chieti, Italy; daniela.novembre@unich.it; 2 Department Mineralogia, Petrologia i Geologia Aplicada, Universitat de Barcelona, 08028 Barcelona, Spain; 3 Dipartimento di Scienze Psicologiche, della Salute e del Territorio, Università degli Studi “G. D’Annunzio”, Via dei Vestini 30, 66013 Chieti, Italy; lucia.marinangeli@unich.it (L.M.); a.tangari@unich.it (A.C.T.); g.rosatelli@unich.it (G.R.); 4 Department of Pharmacy, University of Chieti-Pescara “G. d’Annunzio”, Via dei Vestini, 6100 Chieti, Italy; michele.ciulla@unich.it (M.C.); pietro.diprofio@unich.it (P.d.P.)

**Keywords:** zeolites, rice husk ash, X-ray diffraction, synthesis, NaA LTA Zeolite

## Abstract

The present work deals with the hydrothermal synthesis of a Na-A (LTA) zeolite using rice husk as a starting material. The focus was on defining the most favorable conditions for the synthesis of zeolite Na-A from rice husk in order to economize on both energy (i.e., synthesis temperatures) and reaction time and to enlarge the field of the pure and isolated synthesized phase. Four sets of experiments were carried out at environmental pressure temperatures varying from 40 °C to 85 °C with a SiO_2_/Al_2_O_3_ ratio from 1.75 to 3.5. Optimal conditions for crystallization of the Na-A zeolite from rice husk were reached at 60 °C with a SiO_2_/Al_2_O_3_ ratio of 1.75. Sixty degrees Celsius represents the minimum known temperature used for the synthesis of NaA zeolite from rice husk. The products of synthesis were characterized by X-ray diffraction, scanning electron microscopy, infrared and Raman spectroscopy. The purity of the synthesized zeolite is verified here for the first time through quantitative phase analysis using the combined Rietveld and reference intensity ratio methods.

## 1. Introduction

Rice husk (RH) is an agricultural waste available in rice-producing countries. The potential global RH production is estimated at 21 million tons per year [1]. Generally, much of the husk is burnt or dumped as waste; sometimes, it is converted into end products such as feedstock [2] and adsorbents [3] or is used as a resource of combustion for electricity generation in those countries dependent on imported oil for their energy needs [4]. In many cases, it is burned openly, causing environmental and health problems, especially in poor and developing countries [5]. In some cases, RH is burned in the boiler of various industries to produce RH ash (RHA) which contains 96–99 wt% of amorphous SiO_2_ [6]. RHA can represent a cheap alternative for silica for many industrial uses [7], being a highly reactive reactant for many chemical processes. Barthey et al. [8] used RH silica as a raw material for producing solar-grade silicon (Si-GS) and Johar et al. [9] produced mullite for the ceramic industry starting from RH. RH was also used as an alternative source of silica to synthesize zeolites [10,11,12,13,14,15,16,17,18,19]. Zeolites are crystalline aluminosilicates characterized by an open three-dimensional framework structure built of SiO_4_ and AlO_4_ tetrahedra linked by sharing oxygens to form regular intracrystalline cavities and channels of atomic dimensions [20]. The ion exchange and the selective adsorption properties of zeolites are at the base of several industrial uses. Some of them deal with radioactive wastewater treatment, sewage effluent treatment, agricultural-wastewater treatment [21], etc.

Among low-silica sodium zeolites, the NaA zeolite deserves attention. This zeolite is characterized by two cage types in its lattice: the β-cage (sodalite cage) and α-cage [22]. Eight sodalite cages are connected through double four-membered rings to create a large α-supercage in the middle of eight-membered rings [23]. The Si/Al ratio of 1 confers to the structure a high cation exchange capacity. The most common application is in the field of water detergency. NaA is in fact largely used by the detergent industry in countries with restrictive environmental regulations; it substitutes Na tripoliphospate (NTP), one of the main pollutants responsible for the eutrophization of superficial waters [24]. There are also studies on the absorption of methane [25], carbon dioxide [26], ethylene and propilene [27] by Zeolite NaA. It is also used in the dehydration of ethanol [28] and as an antimicrobial material when exchanged with silver ions [29].

Zeolites are generally synthesized from solutions of sodium silicate and sodium aluminate [30] or from natural materials. The nature of the raw materials strongly influences the purity and quality of the synthesized zeolites [31]. Zeolite synthesis has been performed using different kinds of natural precursors such as kaolinite [32,33,34,35]; bentonite [36,37]; smectite [38]; mordenite [39]; halloysite [40,41]; tripolaceous and volcanic rocks [21,42] and industrial waste materials [43,44,45]. Among waste materials, RH was also used in the synthesis of low-silica sodium zeolites [6,46,47,48,49,50,51,52,53,54,55].

Nur et al. [46] reported the synthesis of NaA zeolites starting from RH; crystallization is performed at 100 °C in 5 h but there is a tendency for formation of amorphous phases in the NaA zeolite. Petkowicz et al. [6] and Bhavornthanayod et al. [4] reported the synthesis of a zeolite A membrane from RHA at 100 °C in 4 h. Yusof et al. [47] established the conversion of RH in a NaA-type zeolite at 100 °C in 6 h. Azizi et al. [48] used microwave-assisted and hydrothermal conventional heating to synthesize a NaA zeolite from RH; after 2 h of microwave heating, the mixture is conventionally hydrothermally treated at 180 °C for 16 h. These authors stated that the combined hydrothermal–microwave technique improves the kinetics in the synthesis of zeolites; however, this is highly questionable given the previous works cited before [6,46,47]. Moreover, the X-ray diffractometric spectrum of NaA lacks the characteristic peaks at 7.10° 2 theta typical of this zeolite [56]. In 2011, Tan et al. [49] reported the synthesis of NaA from RHA at 100 °C in 5 h. More recently, nanocrystals of NaA ranging from 50 to 120 nm were synthesized by Ghasemi and Younesi [50] at room temperature with 3 days aging and without any organic additives. The same authors reported the synthesis of nanocrystals of NaA, ranging from 40 to 120 nm, obtained at 40 °C with 18 h aging [51]. Tepamat et al. [52] synthesized Na-A zeolites from rice husk at 100 °C in 8 h. Ahmedzeki et al. [53] reported the synthesis of NaA zeolites starting from aluminum cans and rice husk; synthesis was performed at 100 °C in a day. Wang et al. [54] synthesized NaA zeolites by the hydrothermal method with seed technology at a temperature of 100 °C for 12 h, while Madhu et al. [55] obtained NaA zeolites from rice husk at 100 °C in 8 h.

As can be seen, apart from the works of Ghasemi and Younesi [50,51] regarding the synthesis of nanosized crystals of NaA zeolites, all the past literature indicates 100 °C as the best lowest temperature at which a zeolite crystallizes.

Considering the previous literature, this work aims to investigate the best condition for the synthesis of NaA zeolites starting from RH. In this paper, we present the results of research carried out to define the most favorable conditions to economize both energy (i.e., synthesis and calcination temperatures) and reaction time. In particular, the work aims on the one hand to lower the rice calcination temperatures below 600 °C and on the other to reduce the synthesis temperatures below 100 °C. The influence of the SiO_2_/Al_2_O_3_ ratios is also investigated.

In recent decades, there has been a renewed interest in NaA zeolites and there has been an increase in the number of scientific publications on the synthesis and applications of this zeolite, as highlighted by Collins et al. [23]. Although the bibliography attests to a wide development of innovative methodologies aimed at the synthesis of this mineral starting from rice husk, it must be said that no industrial transfer followed any protocol. Collins et al. [23] find reasons for this in terms of the manufacturing cost and development of new markets. In our opinion, one of the reasons for this may probably be the lack of quantitative characterizations on the purity of the synthesized products; as is known, one of the essential requirements of the industry is at least 90% purity of the products obtained. Regarding this, none has quantitatively characterized the product of the synthesis yet. The identification of the zeolite peaks in the diffraction spectrum is not sufficient for defining the experimental protocol as successful. What does matter is the estimation of the amorphous phase and/or unreacted reagents in the final product. This is of crucial importance for useful minerals such as zeolites whose usage strongly depends on the purity of powders, which drives their efficacy in technological applications. Alongside a complete spectrum of characterization of the synthesized powders, a quantitative analysis of them will allow us to determine the contribution of the amorphous component and/or other unreacted phases.

This will help to accurately define the purity of the synthesis powders and will pave the way for an industrial transfer of the synthesis protocol.

## 2. Results and Discussion

**Figure 1:**
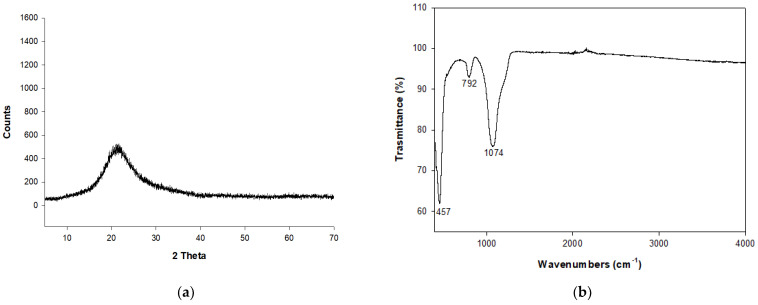
(**a**) XRPD pattern of RHA. (**b**) IR analysis on RHA.

Figure 1a reports the X-ray Powder Diffraction (XRPD) spectrum of RHA, revealing an amorphous character, as is evident from the absence of peaks and the presence of the hump around the 20° of 2 theta.

Figure 1b shows the results of infrared (IR) analysis on RHA. There is a strong band at 1074 cm^−1^, a medium band at 792 cm^−1^ and another strong and narrow band at 457 cm^−1^. The vibration at 1074 cm^−1^ can be assigned to the asymmetric stretching vibrations of tetrahedral SiO_4_, while the peak at 792 cm^−1^ is related to the symmetric stretching of SiO_4_ tetrahedra. The signal at 457 cm^−1^ is associated with the Si-O bending mode. Data are coherent with findings from Petkowicz et al. [6] that indicate the presence of three peaks at 1099, 803 and 464 cm^−1^; also, Yusof et al. [47] report vibrations in agreement with our data, located at 1102, 804 and 470 cm^−1^, respectively.

The chemical composition of RHA is reported in Table 1, resulting in a SiO_2_ content of 98.50 wt%.

**Figure 2:**
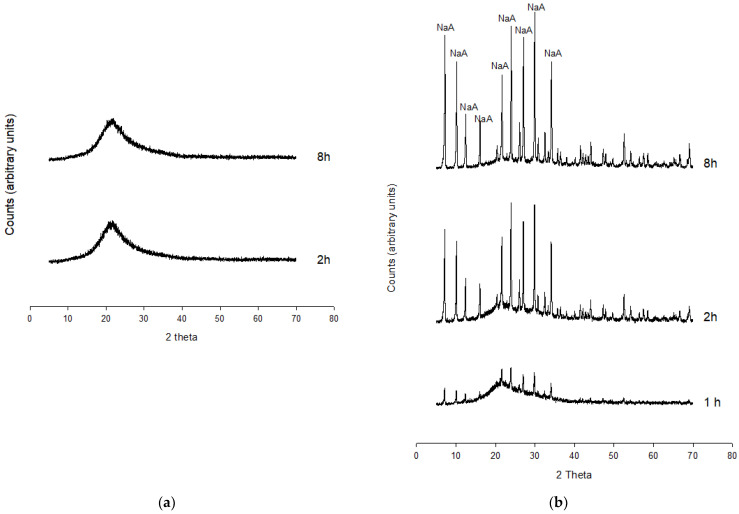
XRPD data. (**a**) XRPD pattern of Experiment 1, (**b**) XRPD pattern of Experiment 2.

XRPD analysis reveals the absence of peaks in the spectrum of Experiment 1 in the time interval 2–8 h, thus resulting in amorphous composition (Figure 2a).

The NaA zeolite is the main phase synthesized in Experiment 2 (Figure 2b). The first appearance is in fact attested at 1 h; the peak intensity increases over time, and the crystallization climax is reached after 8 h.

**Figure 3:**
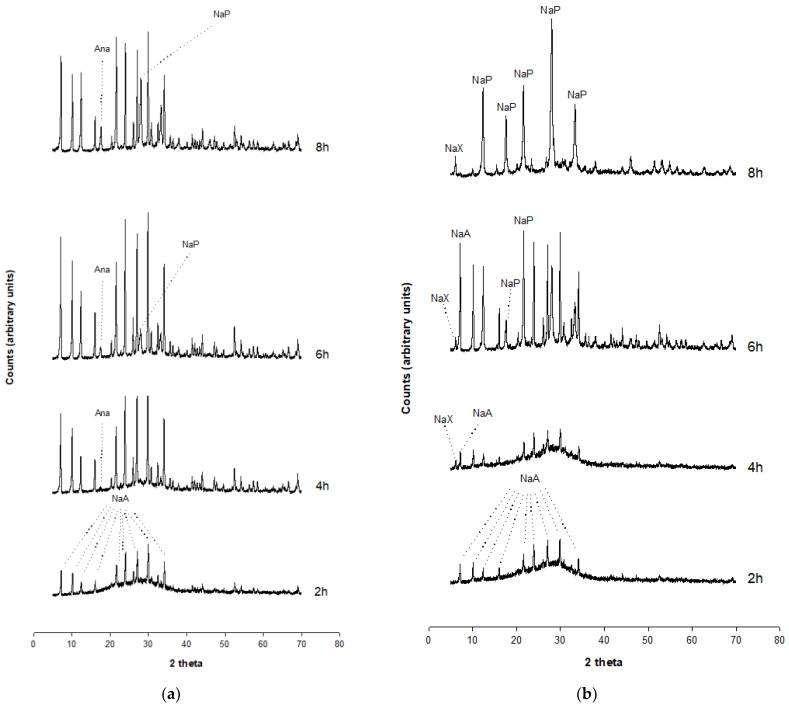
XRPD data. (**a**) XRPD pattern of Experiment 3, (**b**) XRD pattern of Experiment 4.

The beginning of the crystallization of the NaA zeolite is observed as an isolated phase at 2 h in Experiment 3 (Figure 3a); the intensity of the peaks increases after 4 h but the phase is no longer isolated, given the appearance of Analcime (ANA) at 4 h; then, NaA is associated with ANA and NaP in the time interval 6–8 h.

NaA crystallization starts as an isolated phase at 2 h in Experiment 4 (Figure 3b). The phase is associated with NaX at 4 h. Appearance of NaP is registered at 6 h, thus resulting in the overlapping of NaA, NaX and NaP. Disappearance of NaA is evidenced at 8 h, testifying to the progressive replacement of the NaA zeolite by the NaX and NaP.

**Figure 4:**
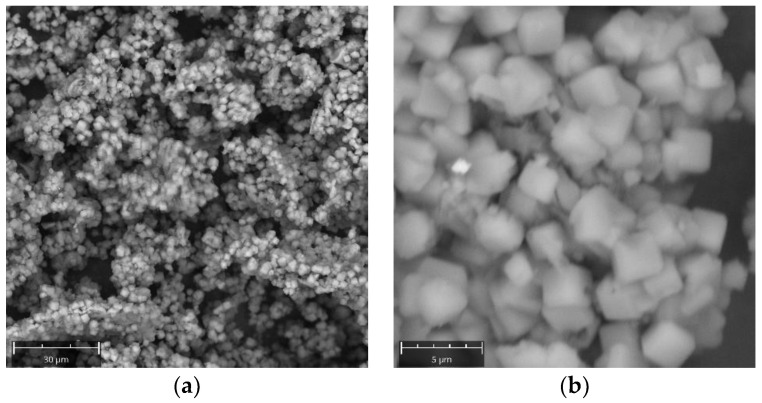
SEM images of NaA zeolite crystals for Experiment 2. (**a**) 6 h, (**b**) 8 h.

All synthetic powders were analyzed by a Scanning Electron Microscope (SEM). Figure 4a,b report Scanning Electron Microscope (SEM) images of NaA crystals from Experiment 2 at 6 and 8 h, respectively. It results in a cubic morphology of the crystals and an average maximum length of crystals of around 3–4 microns.

**Figure 5:**
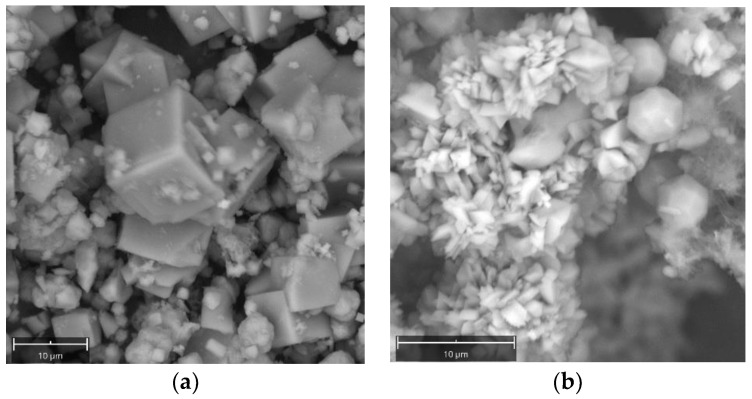
SEM images of zeolite crystals for Experiment 3. (**a**) 4 h, (**b**) 8 h.

Figure 5a,b report SEM images of crystals from Experiment 3. Figure 5a reports crystals of NaA at 4 h, while Figure 5b shows replacement of NaA by rounded euhedral crystals of ANA and acicular crystals of NaP at 8 h.

**Figure 6:**
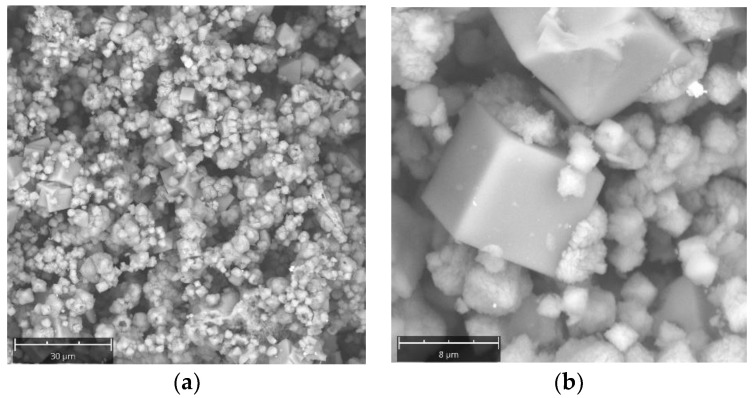
SEM images of zeolite crystals for Experiment 4. (**a**) 4 h, (**b**) 8 h.

Figure 6a,b report SEM images of crystals from Experiment 4. A mix of morphologies associated with different phases are observed, though their identification is difficult due to the low crystalline grain; it can be stated that it deals with NaX and NaP species, as determined by XRD analyses. Among these, characteristic NaA cubic crystals are evident, with an average size of 10 microns.

The results of XRPD and SEM analyses indicate that Experiment 2 achieves the best result in obtaining the NaA zeolite as an isolated phase. Sixty degrees Celsius can be defined as the lowest temperature value at which the NaA zeolite crystallizes as an isolated phase, as achieved in Experiment 2. When the SiO_2_/Al_2_O_3_ ratio is increased to the value of 3.5 at the same temperature (60 °C) in Experiment 4, good results are not obtained, given the overlap of the phases.

The increase in the ratio SiO_2_/Al_2_O_3_ from 1.75 to 3.5 in fact makes the stability of the NaA zeolite unfavorable, delimiting its stability field at 6 h after the start of the synthesis run. The NaA zeolite is quickly consumed and replaced at 8 h by NaP and NaX, which are phases characterized by a higher structural silica content, just as observed by Breck [30].

For this reason, further characterizations were conducted on the sample at 8 h in Experiment 2.

The results of the quantitative phase analysis (QPA) are illustrated in Table 2. The cell parameters of NaA are refined with cubic symmetry, space group *Fm-3c*. The results of the Rietveld refinements provide cell values that are in good agreement with the structural model proposed by Gramlich and Meier [57]. It results in 93.5% of zeolitic phase achieved at 8 h.

**Figure 7:**
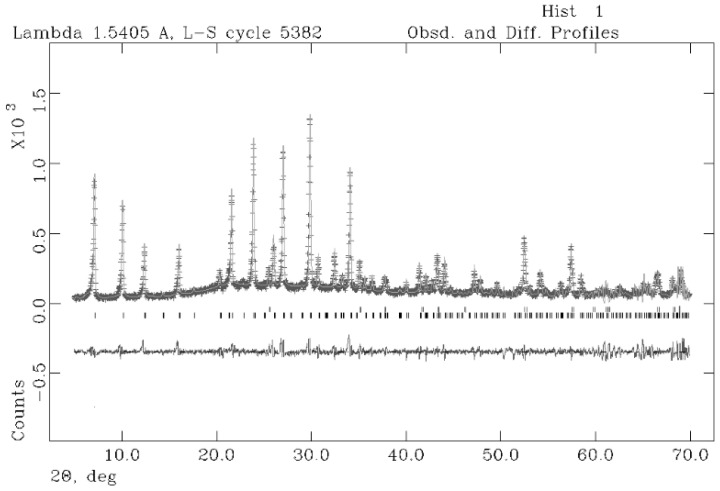
Rietveld refinement plot: observed (+) and calculated profiles and difference plot for NaA zeolite (8 h, Experiment 2) and corundum NIST 676a with tick marks at the position of the Bragg peaks. From the bottom: NaA zeolite, corundum NIST 676a.

The observed and calculated profiles and difference plots for NaA and corundum NIST 676a are reported in Figure 7, showing very good agreement between the experimental data and the structural model used [57].

Using the BET method, the obtained zeolite had a specific surface area of 6.10 m^2^/g, an average pore volume of 0.0029 cm^3^ g^−1^ and a pore size of 19.3959 Å. These values are relatively low, being coherent with data expected for this zeolite [6].

**Figure 8:**
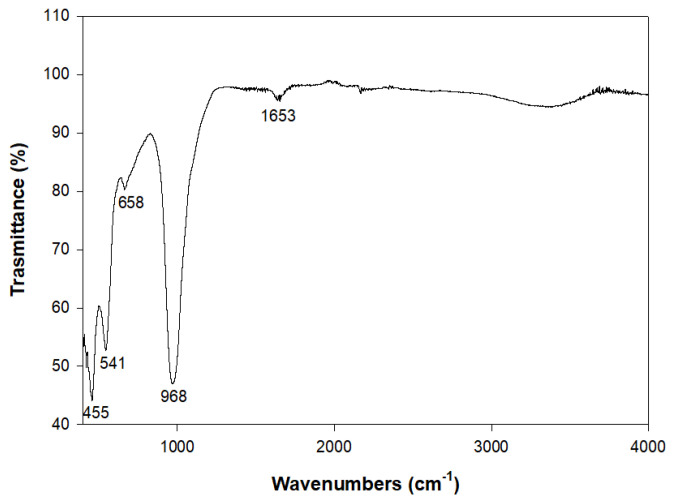
IR spectrum of the sample at 8 h—Experiment 2.

Figure 8 illustrates the results of the IR analysis. The main band is located at 968 cm^−1^ and is related to the region of the asymmetric stretch. The value obtained is consistent with that known for the NaA zeolite [58], with reported values of 995 cm^−1^ for the main band. In the 750–650 cm^−1^ spectral zone, there is a band at 658 cm^−1^ associated with the region of the symmetric stretch.

In the 650–500 cm^−1^ spectral region, relative to the double-ring bond’s vibration zone, there is a band at 541 cm^−1^. In the 500–420 cm^−1^ sector, related to the deformation vibrations of the O-T-O bond, there is evidence of a band at 455 cm^−1^. The bands are in good agreement with those found by Flaningen et al. [58], Fotovat et al. [44] and Yusof et al. [47] for the NaA zeolite.

**Figure 9:**
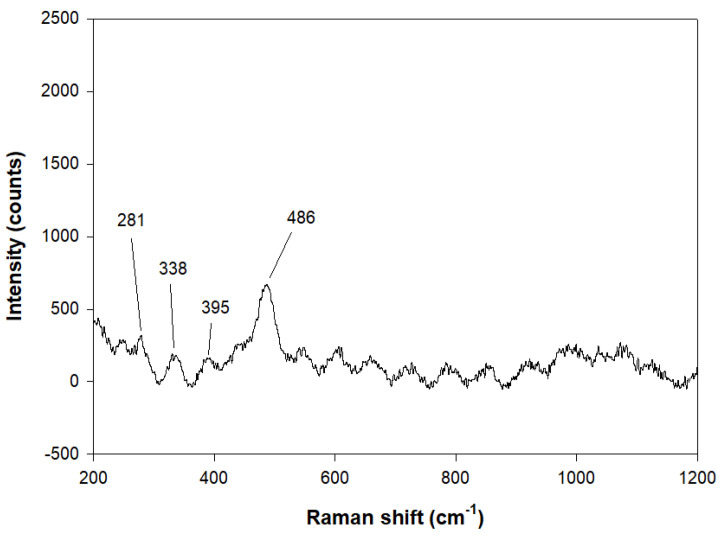
Raman spectra of the sample at 8 h—Experiment 2.

Figure 9 reports the Raman spectrum for the sample at 8 h of Experiment 2. The strongest band at 486cm^−1^ is related to the bending mode of four-membered Si-O-Al rings as reported by No et al. [59]. The bands at 338 and 395 cm^−1^ are attributed to the bending mode of six-membered Si-O-Al rings as indicated by Dutta et al. [60]. The band at 281 cm^−1^ is attributed to the bending mode of the eight-membered rings of the zeolite, just as observed by Yu et al. [61].

## 3. Materials and Methods

The RH sample coming from NE Spain was triturated, washed with water and dried at 105 °C for 24 h. Then, it was subjected to calcination with the following procedure: aliquots of RH were placed in open porcelain crucibles which were heated in a BE43N BICASA furnace (Bernareggio Italy), to the calcination temperature (550 °C). The heating rate of the sample was 1.5 °C s^−1^. Once the calcination temperature was reached, the crucibles were left in the furnace for 5 h and then removed and cooled at room temperature. Following the calcination treatment, an RH ash (RHA) was obtained. The composition of RHA major elements was determined by X-ray fluorescence (XRF), by means of a Sequential X-ray WDXRF, Panalytical (Malvern, UK), Axios PW 4400/40 sequential spectrophotometer at Centres Cientifics i Tecnològics de la Universitat de Barcelona (CCiT-UB). The analysis strategy included the production of 3 fused pearls (original, duplicate and cleaning pearl in a Pt crucible and collector Pt dish, using LiI as a viscosity corrector) at a ratio dilution sample/lithium tetraborate 1/20. The high silica content of RHA represented a problem, taking into account the range of composition of the international standards available for the calibration curve. This was overpassed with an ad hoc procedure consisting of mixing 1:2 and 1:3 proportions of international standards of basaltic composition (kindly provided to DG for Geological Survey of Japan) with the RHA, obtaining results in the calibration range rhyolite–andesite of the instrument. Since light elements, and especially Na, in low contents (i.e., lesser than 2.5% Na_2_O) [62] are a source of trouble, this was solved with an in-house calibration laboratory with internal standards also previously analyzed by AAS [63]. The spectrometer was calibrated using a set of more than 60 international standards [64,65].

The NaOH and NaAlO_2_ used in the synthesis protocol were purchased from Riedel-de Haën (Honeywell Riedel-de Haën, Bucharest, Romania). The purity of the reagent was 99%. Zeolitic synthesis was operated under hydrothermal conditions and environment pressure by the mixing of silicatic and aluminatic solutions.

The silicatic solution was prepared with the following procedure: 0.95 g of the obtained RHA was dissolved in a 29cc NaOH 8 % solution (2M). The aluminatic solution was obtained as follows: 1.38 g NaAlO_2_ was added to 29cc NaOH 8 % solution. The silicate solution was gradually mixed with aluminatic solution in the ratio of 1:1 for Experiments 1, 2 and 3 and 1:0,5 for Experiment 4. The initial mixtures had the following composition: 1.75 SiO_2_–1.00 Al_2_O_3_–5.46 Na_2_O–349 H_2_O (for Experiments 1, 2 and 3) and 3.50 SiO_2_–1.00 Al_2_O_3_–7.69 Na_2_O–536 H_2_O (for Experiment 4). The mixtures were homogenized for two hours with a magnetic stirrer.

Then, they were put inside stainless-steel hydrothermal reactors and heated at 40 °C–60 °C and 85 °C. In Table 3, the experimental conditions are reported, indicating the SiO_2_/Al_2_O_3_, Na_2_O/SiO_2_ and H_2_O/Na_2_O ratios of the starting solutions and the temperatures of synthesis.

**Figure 10:**
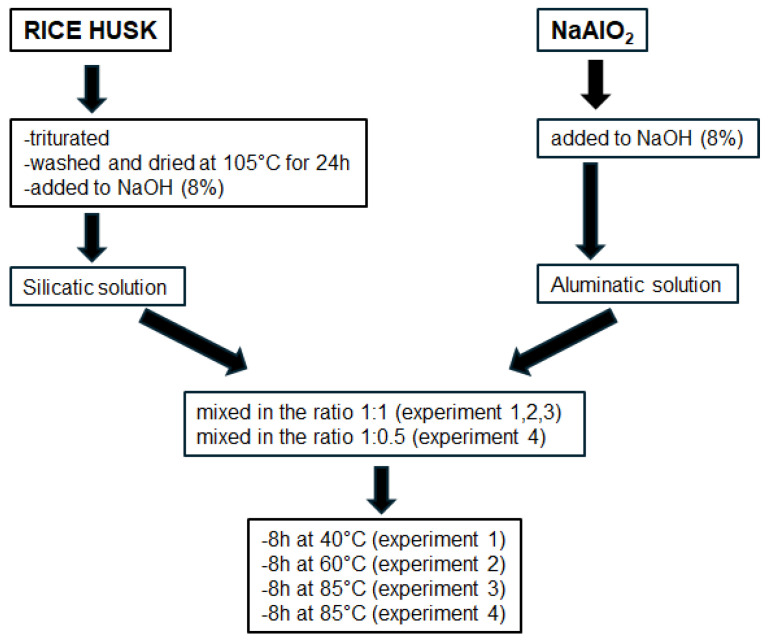
Scheme of experimental protocol.

Figure 10 shows the scheme of the experimental protocol.

The synthesis processes were proved by step-by-step XRD through periodic sampling. Samples obtained at different intervals were filtered from the solution, thoroughly washed with distilled water and oven dried at 40 °C for one day. Samples prepared as smears on glass slides were subjected to XRD analysis [35].

RHA and the products of synthesis were analyzed by powder X-ray diffraction (XRPD); the instrument was a RIGAKU (Osaka, Japan); “MiniFlex II” operating with a Bragg–Brentano geometry (CuKa = 1.518 Å, 30 kV, 15 mA, 5–70° 2 theta scanning interval, step size 0.02° 2 theta, data acquisition speed of 0.033°/s). Identification of zeolites and relative peak assignment were performed with reference to the following JCPDS code: 71-0962 for NaA, 00-019-1180 for ANA, 71-0962 for NaP and 63231-69-6 for NaX. Both the crystalline and amorphous phases in the synthesis powders were estimated using quantitative phase analysis (QPA) applying the combined Rietveld and reference intensity ratio (RIR) methods; corundum NIST 676a was added to each sample, amounting to 10% (according to the strategy proposed by Novembre et al. [66,67]) and the powder mixtures were homogenized by hand-grinding in an agate mortar. Data for the QPA refinement were collected in the angular range 5–70° 2 theta with steps of 0.02° and 10 s step^−1^, a divergence slit of 0.5° and a receiving slit of 0.1 mm.

Data were processed with the GSAS software 3.0 [68] and the graphical interface [69] starting with the structural models proposed by Gramlich and Meier [58] for NaA. The following parameters were refined: background parameters, zero shift, cell parameters and peak profiles [70,71,72,73].

Morphological analyses were obtained by means of scanning electron microscopy Phenom XL SEM–EDX (ThermoFisher Scientific, Dartford, UK); powders were analyzed according to the method explained in Novembre et al. [21] including dehydration of environmental moisture, mounting on auto adhesive stub, and metal coating. Due to the great capacity of water uptake of zeolites and the well-known disturbances in SEM observations, a double coating with graphite and vaporized Au was chosen. The instrumental operative conditions were as follows: high vacuum, accelerating voltage of 15 kV and 2–15 μm beam diameter.

The specific surface and porosity were obtained by applying the BET (Brunauer–Emmett–Teller) method with N_2_ using a Micromeritics (Norcross, GA, USA) ASAP2010 instrument (operating from 10 to 127 kPa).

IR spectra were obtained using a Shimadzu IRAffinity-1S FTIR spectrophotometer (Shimadzu Italia S.r.l., Milan, Italy) equipped with a sealed and desiccated interferometer, a DLATGS (Deuterated Triglycine Sulphate Doped with L-Alanine) detector and a single reflection diamond ATR crystal (QATR 10, Shimadzu Italia S.r.l., Milan, Italy). FTIR spectrum was recorded in the range from 4000 to 400 cm^−1^ co-adding 45 interferograms at a resolution of 4 cm^−1^ with Happ–Genzel apodization. The ATR crystal was carefully cleaned before each analysis, a background was recorded for each sample and the measurements were performed in triplicate [74].

Spectra manipulation was carried out with the software LabSolution IR version 2.27 (Shimadzu Italia S.r.l., Milan, Italy).

Raman spectrum of NaP-1was obtained by confocal and high-performance Raman microscope XploRA PLUS (Horiba. Kyoto, Japan) with deep-cooled CCD detector technology. LabSpec 6.6.1.14 (Horiba, Japan) was employed to control, optimize and process the acquired data. Furthermore, data were processed through Origin 8.5 to optimize the results. Analysis was performed in the range of 50–1700 cm^–1^ and with an 1800-line/mm grating.

## 4. Conclusions

Silica extracted from rice husk, a highly available and economic agricultural waste, is used instead of pure chemicals in the synthesis of NaA zeolites.

Rice husk is calcined at 550 °C. Four sets of experiments were carried out at environmental pressure and hydrothermal conditions; the temperatures and SiO_2_/Al_2_O_3_ ratio varied from 40° to 85 °C and 1.75 to 3.5, respectively.

No crystallization of zeolites was attested at 40 °C with a SiO_2_/Al_2_O_3_ ratio of 1.75.

The optimal conditions for crystallization of Na-A zeolites were reached in Experiment 2, at 60° with a SiO_2_/Al_2_O_3_ ratio of 1.75. Na-A growth was visible at 2 h and peak intensities increased, reaching the maximum values at 8 h.

The coeval presence of zeolitic phases (Na-A, NaP and ANA) was tested in Experiment 3 at 85 °C with a SiO_2_/Al_2_O_3_ ratio of 1.75.

When increasing the SiO_2_/Al_2_O_3_ ratio to 3.5 at 60 °C in Experiment 4, the Na-A stability field diminished and NaP and NaX formation was favored.

Good agreement with bibliographic data was obtained for the infrared and Raman behavior of the NaA zeolite synthesized in Experiment 2 at 8 h. When our experimental protocol is compared with the cited literature, a clear improvement is evident. The calcination temperature of rice husk was reduced from 700 °C to 550 °C, while the synthesis temperature was reduced from 100 °C to 60 °C. The effective assessment of the degree of success of the experiment is here defined for the first time from calculation by QPA of the percentage of crystallization vs. amorphous material and other impurities. The sample at 8 h of Experiment 2 reached 93.5%.

It must be said that if on the one hand a large amount of bibliography is available which testifies to numerous attempts to valorize the use of rice husk in the synthesis of NaA zeolites, on the other hand no work has been followed by an industrial transfer.

We believe that the results achieved with our work can be considered improvements compared to the past and are encouraging from the perspective of an industrial transfer of the experimental protocol. For the first time in this work, the synthesis of NaA zeolites is recorded at temperatures lower than 100 °C and with rice husk calcination temperatures of 550 °C. With reference to the characterization of the synthesis product, the degree of purity of the powders is defined here for the first time; the obtained value of 93.5% is crucial in defining the degree of success of the experimental protocol when considering that industry requires powders at a purity of at least 90%. We believe that the achievement of more advantageous synthesis conditions from an economic point of view and a precise characterization of the zeolite obtained represents a substantial turning point compared to the previous literature and paves the way for a future industrial transfer of the experimental protocol.

## Figures and Tables

**Table 1:** Percentage of chemical composition of RHA determined by XRF.

SiO_2_	Al_2_O_3_	Fe_2_O_3_	CaO	Na_2_O	MnO	TiO_2_	MgO	P_2_O_5_
98.50	0.26	0.18	0.13	0.20	0.14	0.02	0.14	0.11

**Table 2:** Results of the QPA analyses conducted on sample at 8 h in Experiment 2.

Sample + 10% Corundum Nist 676a	60 °C–8 h
R_wp_	0.18
R_p_	0.15
CHI^2^	2.41
space group NaA	*Fm-3c*
*a* (Å)	24.5932 (0.0035)
% amorphous	8.6 (14)
NaA	91.4 (18)

**Table 3:** Experimental conditions used in the four synthesis runs.

	SiO_2_/Al_2_O_3_ Ratio	Na_2_O/SiO_2_ Ratio	H_2_O/Na_2_O Ratio	T (°C)
1	1.75	3.1	63.9	40
2	1.75	3.1	63.9	60
3	1.75	3.1	63.9	85
4	3.50	2.19	69.7	85

## Data Availability

The original contributions presented in the study are included in the article, further inquiries can be directed to the corresponding author.
